# Incidence, Persistence, and Clearance of Anal Human Papillomavirus Infections Among Adolescent and Young Men Who Have Sex With Men in China: An Observational Cohort Study

**DOI:** 10.1093/ofid/ofag352

**Published:** 2026-08-27

**Authors:** Song Fan, Yingming Wang, Ningjun Ren, Maohe Yu, Lin Ouyang, Huachun Zou

**Affiliations:** School of Public Health, Southwest Medical University, Luzhou, Sichuan, China; School of Public Health, Southwest Medical University, Luzhou, Sichuan, China; School of Public Health, Southwest Medical University, Luzhou, Sichuan, China; Department of AIDS/STD Control and Prevention, Tianjin Center for Disease Control and Prevention, Tianjin, China; Department of AIDS/STD Control and Prevention, Chongqing Center for Disease Control and Prevention, Chongqing, China; School of Public Health, Southwest Medical University, Luzhou, Sichuan, China; Shanghai Institute of Infectious Disease and Biosecurity, School of Public Health, Fudan University, Shanghai, China; Melbourne Sexual Health Centre, Faculty of Medicine Nursing and Health Sciences, Monash University, Melbourne, Australia; Center for AIDS Research (CFAR), School of Public Health, Key Laboratory of Public Health Safety, Ministry of Education, Fudan University, Shanghai, China

**Keywords:** human papillomavirus (HPV), men who have sex with men (MSM), incidence, persistence, clearance, China

## Abstract

**Background:**

High-risk adolescent and young men who have sex with men (MSM) populations in China experience a significant burden of human papillomavirus (HPV) infections, particularly involving HPV6, HPV11, HPV16, and HPV18. This prospective observational study investigated the prevalence, persistence, and clearance of HPV, highlighting the critical need for targeted interventions.

**Methods:**

Adolescent and young MSM across 4 Chinese provinces participated in the UniTest Study. Anal swab samples were collected at baseline and follow-up for HPV detection and genotyping. Sociodemographic and behavioral data were obtained through self-administered questionnaires. Incidence, persistence, and clearance rates were calculated, and potential determinants were analyzed using Poisson regression models.

**Results:**

The overall incidence of any HPV genotype was 42.1 per 1000 person-months (pm), with HPV16 (5.6/1000 pm), HPV6 (4.1/1000 pm), and HPV58 (3.2/1000 pm) being the most common incident genotypes. Persistent infections were observed in 28.0%, with HPV11 (4.5%), HPV6 (4.0%), and HPV16 (3.5%) as the top persistent genotypes. The clearance rate for any HPV genotype was 102.6 per 1000 pm. Participants who sought sex partners online had a higher likelihood of HPV incidence (risk ratio [RR] = 1.543, 95% confidence interval [CI] 1.043–2.282), while those with above-average academic performance had a lower likelihood of HPV incidence (RR = 1.551, 95% CI 1.058–2.274). Compared with Han, other ethnicities were associated with increased HPV clearance (RR = 5.757, 95% CI 1.067–31.067).

**Conclusions:**

This study uncovers a substantial incidence of HPV infections among adolescent and young MSM in China, underscoring the imperative for focused health education and the swift implementation of customized HPV vaccination initiatives. Through the prioritization of preventive strategies and vaccination campaigns, it’s possible to significantly diminish the impact of HPV infections and enhance sexual health outcomes within this at-risk group.

**Trial registration.** Chinese Clinical Trial Registry, www.chictr.org.cn: ChiCTR1900020645.

Human papillomavirus (HPV) has emerged as a crucial factor contributing to the rise in anal cancer incidence, particularly with the prevalence of high-risk (HR) strains [1]. Over the past few decades, there has been a concerning uptick in the occurrence of anal cancer [2, 3]. Men who have sex with men (MSM) face a 15.5-fold higher risk of developing anal cancer compared to their heterosexual counterparts. This heightened risk is attributed to increased susceptibility to HPV infection resulting from anal injury [4–8].

Among adolescent and young MSM, HPV infections are alarmingly prevalent and rising [9–12]. A European and US-based systematic review revealed that 16%−18% of MSM under 25 years of age were infected with HPV 16 [6]. Of particular concern is the prolonged duration of infections and lower clearance rates experienced by adolescent and young MSM, potentially amplifying transmission risks [9, 13]. While some countries have implemented HPV vaccination programs for men or MSM [10, 14, 15], many nations still restrict HPV vaccine administration to females. Consequently, MSM may not fully benefit from the herd immunity conferred by vaccination efforts targeting women [16].

Several cross-sectional studies have characterized HPV genotype prevalence in Chinese MSM populations. For example, Zhou et al reported genotype distribution in MSM samples from Beijing and Shanghai, identifying HPV16 and HPV6 as predominant types [9]. However, these studies are predominantly cross-sectional in design, limiting inference about HPV natural history (incidence and clearance) in this population. Moreover, existing studies have primarily enrolled older MSM (mean age >25 years) or clinical populations (eg, VTC attendees), potentially limiting generalizability to adolescent and young university MSM aged 16-24 years. Our study addresses 3 critical gaps in the literature: (1) Population focus: This is the first prospective study specifically targeting adolescent and young university MSM (aged 16-24 years) in China. This age group has been largely understudied in China's HPV epidemiology literature, despite evidence from other countries indicating exceptionally high HPV acquisition rates in younger MSM cohorts (eg, HYPER study in Australia). (2) Natural history data: While existing studies report HPV prevalence, our study is among the first to prospectively document both HPV incidence (new infections) and clearance rates in Chinese MSM. These natural history parameters are essential for modeling disease burden and evaluating vaccination efficacy. (3) Vaccine-targeted genotypes: We provide detailed analysis of incidence and clearance for genotypes targeted by HPV vaccines currently licensed in China (2-, 4-, and 9-valent formulations), with direct implications for vaccination policy decisions for adolescent MSM in China. These novel contributions position our study as a critical evidence base for informing public health recommendations for HPV prevention in Chinese youth.

## METHODS

### Study Population

This study was conducted as part of the University Student HIV Test Intervention Study (UniTest Study) [18], which recruited adolescent and young MSM from 4 provinces in China (Chongqing, Guangdong, Shandong, and Tianjin) between January and April 2019. The UniTest Study was ethically approved by the institutional review boards at Sun Yat-sen University (approval no. SYSU-SPH2018044), and informed consent was obtained from all participants. Eligible participants were male university students, aged 16 or above, who self-reported having sex with men and tested negative for HIV.

### Recruitment

Participants were recruited from March 2019 to August 2019 through community-based channels in 4 provinces or municipalities in China (Chongqing, Guangdong, Shandong, and Tianjin). Specifically, recruitment strategies included: (1) online advertisements on gay social networking (GSN) applications and social media platforms; (2) outreach through community-based organizations (CBOs) serving MSM, including local LGBT centers and gay rights advocacy groups; (3) offline promotion on university campuses and student organizations; and (4) peer referrals through existing participants. Importantly, recruitment did NOT occur through clinical settings such as sexually transmitted infection (STI) clinics, voluntary testing and counseling centers, or hospitals. This community-based recruitment approach avoided the high-risk selection bias inherent in clinic-based cohorts, which typically enroll individuals already experiencing health concerns or seeking services. Our approach ensured enrollment of a more representative sample of adolescent and young MSM in the general population, including those with no prior history of clinical care or STI diagnosis.

### Eligibility criteria

Eligible participants were biologically male university students aged 16-24 years who self-reported ever having sex with men. Participants could have engaged in insertive-only anal intercourse, receptive-only anal intercourse, or both insertive and receptive anal intercourse; we did not restrict eligibility based on specific sexual roles. All participants were required to be HIV negative at baseline enrollment and provide written informed consent. We explicitly did not exclude participants based on other characteristics, such as lack of healthcare utilization, prior STI history, or sexual role preferences. The rationale for including all MSM (regardless of sexual role) reflects the epidemiology of HPV transmission in MSM: while HPV transmission occurs most efficiently through receptive anal intercourse, insertive partners can also acquire HPV through intact or microabided anogenital epithelium. Our sample composition allows for population-level incidence estimates that are representative of the broader MSM population, though HPV incidence estimates should be interpreted in light of the proportion of participants engaging in receptive anal intercourse.

### Data Collection

Data were collected using a self-administered online questionnaire designed to gather information on sociodemographic factors, including age, ethnicity, education level, major of study, academic performance, dwelling location, and sexual orientation. Additionally, sexual behavior information was collected, such as the age at first anal intercourse, history of engaging in sexual activity under the influence of alcohol or drugs, history of sexually transmitted infections, use of gay dating applications, and the practice of seeking sexual partners online.

### Sexual behavior assessment

Sexual behaviors were assessed using a self-administered online questionnaire administered at baseline and 12-month follow-up. Participants reported detailed information on: (1) age at first anal intercourse (insertive and/or receptive); (2) number and characteristics of male sexual partners over the previous 3 months; (3) condom use practices with regular and casual partners; (4) use of online platforms to seek partners; and (5) meeting venues for sexual partners. Offline meeting venues were categorized as follows:

- Gay bars/nightclubs: establishments specifically catering to gay/MSM clientele with dancing, socializing, and potential sexual encounters.
- Saunas/bathhouses: facilities offering steam rooms, massage services, and private booths with explicit sexual activity.
- Public cruising places: outdoor locations or public facilities (parks and restrooms) known for sexual encounters.
- University campus settings: dormitories, campus grounds, or student social events.
- Private gatherings: parties, friend networks, or home-based social events.
- Participants could report using multiple venue types over the study period.

### Online sex-seeking platforms

Participants reported use of GSN applications and social media platforms to seek male sexual partners. In our study setting, the most commonly used platforms included BlueD, Blued, WeChat, and QQ, which are designed primarily for gay and bisexual men in mainland China. Notably, these platforms support both synchronous contact (real-time, geolocation-based matching of nearby users) and asynchronous contact (message-based communication with remote partners). The availability of both contact types, alongside their regulatory environment in China (less restrictive than in Western Europe and North America), may influence patterns of casual sexual networking and risk behavior among Chinese MSM. This context differs from some Western platforms that may emphasize primarily synchronous “hookup” features; therefore, we conducted a stratified analysis by contact type (synchronous vs asynchronous) to examine their independent associations with HPV incidence (see Results).

### Study incentives and follow-up procedures

All participants received a modest monetary compensation of ¥50 (approximately USD $7-8) for completing each study visit, in addition to free HPV testing and results counseling. This modest incentive was designed to acknowledge participants' time and travel costs without introducing substantial financial inducement. At the 12-month follow-up visit, all participants with uncleared high-risk HPV genotypes (defined as the same genotype positive at both baseline and follow-up) were provided with written and verbal counseling from trained staff. Counseling included: (1) explanation of the natural history of HPV infection and associated disease risks; (2) referral to local CDC clinics or certified STI services for repeat HPV screening and regular sexual health counseling; (3) safer sex education emphasizing condom use and partner reduction; and (4) HPV vaccination information and facilitated referral to vaccination providers if eligible.

### HPV Detection and Genotyping

Anal swab samples were collected from participants for HPV detection and genotyping through DNA analysis. The Hybribio PCR HPV Genotyping Test (Hybribio Biochemical Co., Ltd, Chaozhou, China) was used to determine the distribution of HPV types in the samples. This test amplifies DNA for a total of 37 types, including 13 HR genotypes (16, 18, 31, 33, 35, 39, 45, 51, 52, 56, 58, 59, and 68) and 24 low-risk (LR) genotypes (6, 11, 26, 34, 40, 42, 43, 44, 53, 54, 55, 57, 61, 66, 67, 69, 70, 71, 72, 73, 81, 82, 83, and 84) commonly found in the anogenital area [19, 20]. This comprehensive approach allows for the identification of both HR and LR HPV types, providing valuable data on the prevalence and distribution of various HPV strains among this specific population in China.

### Statistical Analyses

#### Definitions

HPV positive: Tested positive for any HPV type.

High-risk HPV positive: Tested positive for one or more HR HPV types.

Low-risk HPV positive: Tested positive for one or more LR HPV types.

Vaccinal HPV-type infection: Tested positive for HPV types included in the 9-valent (9V), 4-valent (4V), or 2-valent (2V) HPV vaccines.

Incident HPV infection: Newly identified HPV infection in participants who tested negative at baseline.

Cleared HPV infection: Absence of previously detected HPV type at follow-up.

Persistent HPV infection: Presence of the same HPV type at baseline and follow-up.

#### Measures and Calculations

Incidence rate: Number of incident HPV infections divided by the total person-months (pm) of follow-up.

Clearance rate: Number of cleared HPV infections divided by the total pm of follow-up.

Persistence rate: Number of persistent HPV infections divided by the total number of HPV infections at baseline.

Person-months: Duration from baseline participation to the end of follow-up for each participant.

Transmission probability per susceptible individual: Number of incident infections divided by the number of sex partners presumed to be infected.

#### Statistical Methods

Descriptive statistics employed median and interquartile range (IQR) for continuous variables, while categorical variables were summarized using frequency tables. Fisher's exact test and χ^2^ test compared sociodemographic and behavioral characteristics between participants with complete follow-up and those tested only at baseline. Poisson regression evaluated the relationship between sociodemographic and behavioral factors and the incidence and clearance of HPV infection. R version 4.1.0 was used for all statistical analyses. *P* value of <.05 was considered statistically significant.

## RESULTS

### Population Characteristics

A total of 200 adolescent and young MSM completed both baseline and follow-up HPV tests. The median age of participants was 21 years (IQR: 20–22), with a majority identifying as Han Chinese. Notably, 82.5% held a bachelor's degree, and their majors primarily fell within the fields of science and technology (48.0%) and literature and history (20.5%).

Regarding sexual behavior, over half of the participants sought sexual partners online (52.5%), and nearly all utilized gay social networking applications (98.5%). Additionally, a significant proportion reported engaging in drunken sex (76.0%) and recreational drug use during sexual activity (66.0%).

Regarding age at first anal intercourse, the mean (SD) age at first anal receptive intercourse was 18.7 (2.1) years (median: 18, IQR: 17−20 years). Approximately 72.5% (n = 145) of participants reported engaging in receptive anal intercourse at some point in their sexual history. This early age at sexual debut underscores the critical window for preventive HPV vaccination prior to sexual exposure.

Among offline sex-seeking venues, the most frequently reported were university campus settings (38.5%, n = 77), followed by private gatherings (32.0%, n = 64), gay bars (18%, n = 36), public cruising places (9.5%, n = 19), and saunas/bathhouses (7.0%, n = 14).

For a comprehensive overview of the sociodemographic and behavioral characteristics of all participants, please refer to Supplementary Table 1.

### Recruitment characteristics

Of the 200 participants with complete follow-up data, 120 (60%) were recruited through online channels (GSN applications and social media), while 80 (40%) were recruited through offline community-based channels (university campus outreach, CBO partnerships, and peer referrals). In stratified analyses, the incidence of any HPV genotype was 44.2 per 1000 pm (95% CI: 36.8-52.9) among online-recruited participants and 39.9 per 1000 pm (95% CI: 30.1-52.9) among offline-recruited participants, with no statistically significant difference (*P* = .62). This finding suggests that HPV incidence patterns were similar regardless of recruitment channel, supporting the validity of combining both groups for primary analyses.

### Incidence, Clearance, and Persistence of Anal HPV in Adolescent and Young MSM

#### Incidence

The overall incidence of any HPV infection was 42.1 per 1000 pm of follow-up. This rate varied depending on the type of HPV, with HR HPV infections occurring at a rate of 21.5 per 1000 pm and LR HPV infections occurring at a rate of 20.6 per 1000 pm. Additionally, HPV types included in the 9V, 4V, and 2V vaccines were found at rates of 18.7, 13.5, and 7.7 per 1000 pm, respectively. Among the specific HPV types, HPV16, HPV6, HPV58, HPV67, and HPV18 were responsible for the most incident infections.

#### Clearance

The clearance rate for any HPV infection was 102.6 per 1000 pm, demonstrating a relatively high rate of infection clearance in this population. HR HPV infections cleared at a rate of 67.0 per 1000 pm, while LR HPV infections cleared at a rate of 63.6 per 1000 pm. Clearance rates for HPV types included in the 9V, 4V, and 2V vaccines were 50.9, 41.0, and 49.6 per 1000 pm, respectively. Notably, HPV51, HPV11, HPV61, HPV6, and HPV39 were the last HPV types to be cleared during the study period.

#### Persistence

The persistence rate for any HPV infection was 28.0%, indicating that a significant proportion of infections persisted over the follow-up period. Persistence rates varied depending on the type of HPV, with HR HPV infections persisting at a rate of 12.5% and LR HPV infections persisting at a rate of 26.0%. Human papillomavirus types included in the 9V, 4V, and 2V vaccines exhibited persistence rates of 15.5%, 13.0%, and 4.5%, respectively. Interestingly, HPV11, HPV6, HPV16, and HPV51 were the most persistent HPV types identified in this study.

Table 1 shows the incidence, persistence, and clearance of HPV infection among adolescents and young MSM.

**Table 1.** Incidence, Persistence, and Clearance of HPV Infection Among Adolescent and Young Men Who Have Sex With Men

HPV Genotypes	Incident Infections	Person-Months at Risk	Incidence/1000 Person-Months (95% CI)	Cleared Infections	Person-Months of Infection	Clearance/1000 Person-Months (95% CI)	Persistent Infections (N = 200)	% (95% CI)
Any genotypes	135	3209	42.1 (35.5–49.8)	107	1043	102.6 (85.2–123.0)	56	28.0 (22.2–34.6)
HR genotypes	69	3209	21.5 (16.9–27.3)	52	776	67.0 (50.9–87.5)	25	12.5 (8.6–17.8)
HPV16	16	2848	5.6 (3.3–9.3)	13	361	36.0 (20.1.62.3)	7	3.5 (1.7–7.1)
HPV18	8	3001	2.7 (1.3–5.5)	10	208	48.1 (24.6–89.2)	2	1.0 (0.3–3.6)
HPV31	3	3154	1.0 (0.3–3.1)	2	55	36.4 (6.3–136.1)	1	0.5 (0.1–2.8)
HPV33	0	3141	0.0 (0.0–1.5)	3	68	44.1 (11.5–131.9)	0	0.0 (0.0–1.9)
HPV35	3	3184	0.9 (0.2–2.9)	1	25	40.0 (2.1–223.2)	0	0.0 (0.0–1.9)
HPV39	8	3122	2.6 (1.2–5.3)	3	87	34.5 (9.0–104.5)	3	1.5 (0.5–4.3)
HPV45	1	3113	0.3 (0.0–2.0)	5	96	52.1 (19.3–123.0)	1	0.5 (0.1–2.8)
HPV51	5	3022	1.7 (0.6–4.2)	3	187	16.0 (4.1–49.9)	6	3.0 (1.4–6.4)
HPV52	3	3149	1.0 (0.3–3.1)	3	60	50.0 (13.0–148.2)	2	1.0 (0.3–3.6)
HPV56	0	3162	0.0 (0.0–1.5)	3	47	63.8 (16.6–185.6)	1	0.5 (0.1–2.8)
HPV58	10	3158	3.2 (1.7–6.1)	2	51	39.2 (6.8–145.9)	1	0.5 (0.1–2.8)
HPV59	6	3209	1.9 (0.8–4.3)	0	0	…	0	0.0 (0.0–1.9)
HPV68	6	3135	1.9 (0.8–4.4)	4	74	54.1 (17.5–139.9)	1	0.5 (0.1–2.8)
LR genotypes	66	3209	20.6 (16.1–26.3)	55	865	63.6 (48.7–82.5)	52	26.0 (20.4–32.5)
HPV53	2	3131	0.6 (0.1–2.5)	3	78	38.5 (10.0–116.0)	2	1.0 (0.3–3.6)
HPV73	3	3156	1.0 (0.3–3.1)	2	53	37.7 (6.5–140.8)	1	0.5 (0.1–2.8)
HPV82	1	3196	0.3 (0.0–2.0)	1	13	76.9 (4.0–379.1)	0	0.0 (0.0–1.9)
HPV6	12	2910	4.1 (2.2–7.4)	10	299	33.4 (17.1–62.5)	8	4.0 (2.0–7.7)
HPV11	7	2947	2.4 (1.1–5.2)	8	262	30.5 (14.2–61.5)	9	4.5 (2.4–8.3)
HPV34	1	3209	0.3 (0.1–2.0)	0	0	…	0	0.0 (0.0–1.9)
HPV40	5	3066	1.6 (0.6–4.0)	7	143	49.0 (21.6–102.1)	2	1.0 (0.3–3.6)
HPV42	6	3147	1.9 (0.8–4.4)	3	62	48.4 (12.6–143.8)	2	1.0 (0.3–3.6)
HPV43	3	3196	0.9 (0.2–2.9)	1	13	76.9 (4.0–379.1)	0	0.0 (0.0–1.9)
HPV44	2	3168	0.6 (0.1–2.5)	2	41	48.8 (8.5–178.1)	0	0.0 (0.0–1.9)
HPV54	3	3159	0.9 (0.2–2.9)	3	50	60.0 (15.6–175.4)	0	0.0 (0.0–1.9)
HPV55	1	3180	0.3 (0.0–2.0)	2	29	69.0 (12.0–242.2)	1	0.5 (0.1–2.8)
HPV61	2	3078	0.6 (0.1–2.5)	4	131	30.5 (9.8–81.1)	2	1.0 (0.3–3.6)
HPV67	9	3112	2.9 (1.4–5.7)	4	97	41.2 (13.3–108.2)	3	1.5 (0.5–4.3)
HPV69	2	3209	0.6 (0.1–2.5)	0	0	…	0	0.0 (0.0–1.9)
HPV70	2	3209	0.6 (0.1–2.5)	0	0	…	0	0.0 (0.0–1.9)
HPV71	0	3180	0.0 (0.0–1.5)	1	29	34.5 (1.8–196.3)	0	0.0 (0.0–1.9)
HPV72	0	3196	0.0 (0.0–1.5)	1	13	76.9 (4.0–379.1)	0	0.0 (0.0–1.9)
HPV81	2	3185	0.6 (0.1–2.5)	1	24	41.7 (2.2–231–2)	1	0.5 (0.1–2.8)
HPV84	3	3163	1.0 (0.3–3.1)	3	46	65.2 (17.0–189.3)	0	0.0 (0.0–1.9)
Vaccine-preventable genotypes								
9V genotypes	60	3209	18.7 (14.4–24.2)	56	1100	50.9 (39.0–66.0)	31	15.5 (11.1–21.2)
4V genotypes	43	3185	13.5 (9.9–18.3)	41	1001	41.0 (29.9–55.7)	26	13.0 (9.0–18.4)
2V genotypes	24	3105	7.7 (5.0–11.6)	23	464	49.6 (32.4–74.6)	9	4.5 (2.4–8.3)
HPV6/11	19	3185	6.0 (3.7–9.5)	18	561	32.1 (19.7–51.2)	17	8.5 (5.4–12.2)

Abbreviations: 2V, 2-valent vaccines; 4V, 4-valent vaccines; 9V, 9-valent vaccines; CI, confidence interval; HPV, human papillomavirus; HR, high-risk genotypes; LR, low-risk genotypes.

**Figure 1.**
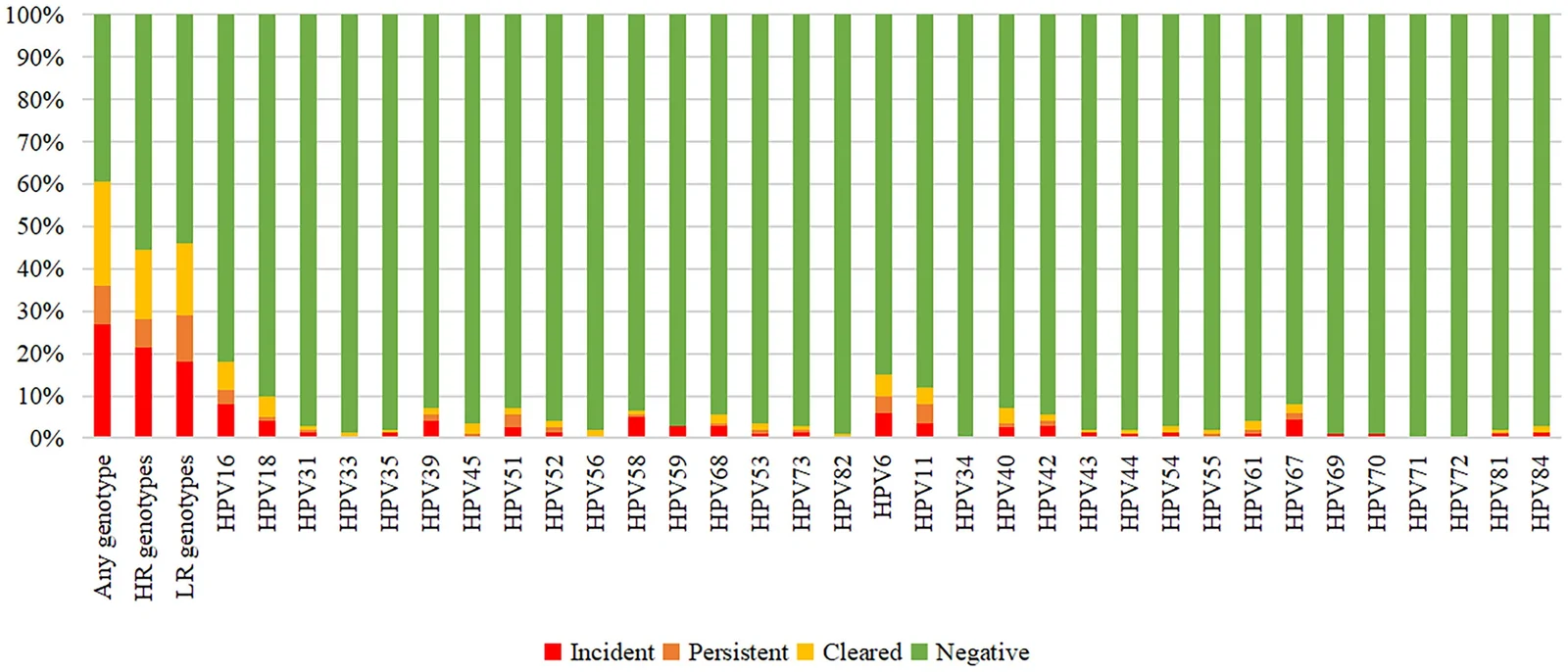
Distribution of incident, persistent, and cleared infections for genotype-specific human papillomavirus (HPV) among adolescent and young men who have sex with men. Negative refers to negative HPV at both visits. HR refers to high-risk genotypes. LR refers to low-risk genotypes.

Figure 1 shows the distribution of incident, persistent, and cleared infections for genotype-specific HPV among adolescent and young MSM.

### Factors Associated With HPV Incidence and Clearance in Adolescent and Young MSM

Among the 105 participants who sought sex partners online, 103 (98.1%) reported using primarily synchronous platforms, while 8 (7.6%) used asynchronous contact methods. In stratified analyses, synchronous online sex–seeking was associated with (RR = 1.54, 95% CI: 1.04–2.28), while asynchronous contact was associated with (RR = 1.12, 95% CI: .65–1.93). Both contact types were associated with elevated HPV incidence compared with offline sex–seeking, though only synchronous contact reached statistical significance.

In the univariate model, several factors were associated with HPV incidence in adolescent and young MSM. Specifically, participants who had a history of recreational drug use during sex, sought sex partners online, and had average or below-average academic performance were more likely to have incident HPV infection. In the multivariable model, after controlling for other variables, seeking sex partners online remained significantly associated with a higher likelihood of incident HPV infection (risk ratio [RR] = 1.543; 95% confidence interval [CI] 1.043–2.282). On the other hand, participants with above-average academic performance were found to be less likely to contract HPV compared to those with average and below-average academic performance (RR = 1.551; 95% CI 1.058–2.274). Compared with Han, other ethnicities were associated with increased HPV clearance (RR = 5.757, 95% CI 1.067–31.067).

Table 2 shows the factors associated with incidence and clearance of any HPV genotypes among adolescent and young MSM.

**Table 2.** Factors Associated With Incidence and Clearance of any HPV Genotypes Among Adolescent and Young MSM

Factors	Incidence (N = 200)				Clearance (N = 85)			
	N	1000 Person-Months	RR	*P* value	n	1000 Person-Months	RR	*P* value
Age at recruitment (years)								
≤21	45	36.1	Ref.		11	21.3	Ref.	
>21	29	33.2	1.040 (0.718–1.506)	0.836	17	30.3	1.540 (0.720–3.292)	0.266
Ethnicity								
Han Chinese	73	34.9	Ref.		26	24.9	Ref.	
Other	1	31.2	0.669 (0.136–3.298)	0.622	2	57.7	5.757 (1.067–31.067)	0.042
Location of participant								
Chongqing	14	26.9	Ref.		7	43.9	Ref.	
Guangzhou	7	18.6	1.030 (0.492–2.153)	0.136	5	29.9	0.975 (0.374–2.538)	0.958
Qingdao	23	37.0	1.710 (1.000–2.926)	0.050	3	11.4	0.353 (0.113–1.102)	0.073
Tianjin	30	53.5	1.542 (0.873–2.726)	0.938	13	27.3	0.726 (0.344–1.532)	0.401
Study level								
Senior/junior college	14	38.1	Ref.		5	21.2	Ref.	
Bachelor's degree	60	34.2	1.001 (0.618–1.623)	0.996	23	27.3	0.547 (0.173–1.732)	0.305
Major								
Literature/history	18	46.3	Ref.		5	23.6	Ref.	
Science/technology	33	31.0	0.716 (0.459–1.116)	0.140	16	30.6	1.753 (0.737–4.169)	0.204
Medicine	6	44.7	1.149 (0.584–2.263)	0.687	1	9.2	0.327 (0.077–1.383)	0.129
Art	9	41.7	1.023 (0.532–1.967)	0.945	3	23.2	0.708 (0.227–2.206)	0.552
Kinesiology/other	8	25.2	0.742 (0.361–1.523)	0.415	3	28.8	0.884 (0.266–2.937)	0.841
Sought sex partner online								
No	27	25.1	Ref.		13	25.4	Ref.	
Yes	47	44.5	1.543 (1.043–2.282)	0.030	15	26.5	0.527 (0.262–1.059)	0.072
Gay app using								
No	2	137.9	1.725 (0.788–3.773)	0.172	1	28.0	3.773 (0.611–23.286)	0.153
Yes	72	34.2	Ref.		27	25.9	Ref.	
Recreational drug use sex history								
No	35	48.9	Ref.		8	23.1	Ref.	
Yes	39	27.7	0.690 (0.468–1.018)	0.061	20	27.4	0.983 (0.499–1.933)	0.959
Drunk sex history								
No	22	47.3	Ref.		9	24.0	Ref.	
Yes	52	31.4	0.726 (0.487–1.083)	0.117	19	27.1	0.827 (0.377–1.811)	0.634
Ever heard of HPV								
No	29	37.5	1.214 (0.860–1.713)	0.270	8	22.8	0.538 (0.250–1.157)	0.112
Yes	45	33.4	Ref.		20	27.5	Ref.	
History of sexually transmitted diseases (STDs)								
Yes	6	29.3	Ref.		5	31.1	Ref.	
No	53	33.7	1.510 (0.797–2.862)	0.206	19	26.0	1.241 (0.379–4.060)	0.624
Unclear	15	43.7	1.627 (0.779–3.401)	0.195	4	21.4	1.282 (0.475–3.643)	0.721
Academic performance								
Above average	51	32.3	Ref.		24	27.7	Ref.	
Average and below	23	42.4	1.551 (1.058–2.274)	0.024	4	19.0	0.468 (0.180–1.214)	0.118
Sexual orientation								
Homosexual	67	38.0	Ref.		28	27.1	Ref.	
Bisexual	5	15.4	0.736 (0.396–1.370)	0.334	0	0.0	…	…
Other	2	54.6	1.340 (0.467–3.843)	0.586	0	0.0	…	…
Age at first anal intercourse (years)								
<18	25	56.3	Ref.		10	26.6	Ref.	
≥18	49	29.2	0.890 (0.622–1.274)	0.525	18	25.7	0.913 (0.422–1.974)	0.816

Abbreviations: 2V, 2-valent vaccines; 4V, 4-valent vaccines; 9V, 9-valent vaccines; HPV, human papillomavirus; HR, high-risk genotypes; LR, low-risk genotypes; MSM, men who have sex with men; RR, risk ratio.

### Recreational drug use during sex

Among the 200 study participants, 132 (66.0%) reported recreational drug use during sexual activity. The most commonly used substances were methamphetamine (popularly known as “ice”; 58%, n = 116) and alkyl nitrites/poppers (28%, n = 56), followed by cannabis (5.0%, n = 10) and synthetic cathinones (4.0%, n = 8). Notably, 32.6% (n = 65) of participants reported using multiple types of drugs during sexual encounters (polysubstance use). In multivariable models, recreational drug use during sex was not significantly associated with HPV incidence after controlling for other factors (RR = 0.690, 95% CI: .468–1.018, *P* = .061); however, the trend toward lower incidence in drug–using participants may reflect confounding by unmeasured factors or the temporal dynamics of drug use and partner change.

**Figure 2.**
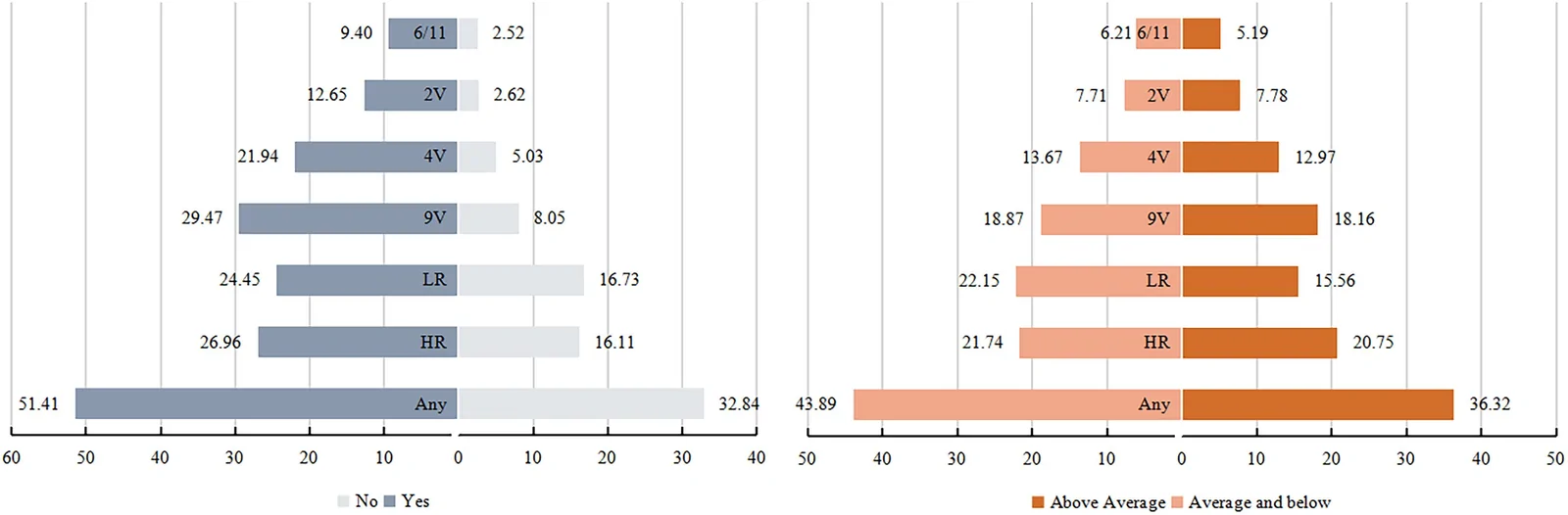
Comparison of human papillomavirus (HPV) incidence rates for various genotype groups among adolescent and young men who have sex with men. *A*, Impact of internet use for seeking sexual partners on HPV incidence. Left bars: used internet for seeking partners. Right bars: did not use internet for seeking partners. *B*, Impact of academic performance on HPV incidence. Left bars: above-average academic performance. Right bars: average or below-average academic performance. HR refers to high-risk genotypes. LR refers to low-risk genotypes. 2V refers to 2-valent vaccines. 4V refers to 4-valent vaccines. 9V refers to 9-valent vaccines.

Figure 2 shows the comparison of HPV incidence rates for various genotype groups among adolescent and young MSM.

## DISCUSSION

The UniTest study stands as a landmark investigation, marking the first exploration of anal HPV incidence, persistence, and clearance among adolescent and young MSM in China. Our research sheds light on the spread and behavior of HPV infections within this demographic, unveiling a significant incidence of HPV infections, especially with HPV6 and HPV16 genotypes. Almost half of the adolescent and young MSM participants were detected with HPV infection either at baseline or follow-up, with the number of infections significantly increasing over the 12-month period. Intriguingly, this heightened incidence was associated with seeking sex partners online and having average or below-average academic performance.

Our study identified a high rate of incident HPV infections among adolescent and young MSM, particularly involving HPV6 and HPV16 genotypes. This finding is consistent with those from a recent observational cohort study conducted in Melbourne, Australia, which highlighted a substantial prevalence of anal, penile, and oral HPV infections in teenage MSM, with HPV16 being notably predominant. The study's estimates of site-specific transmission probabilities per partner for teenage MSM underscore the significant risk this population faces regarding HPV transmission and the critical need for high vaccination coverage in MSM to mitigate these risks [21]. Moreover, our results align with previous cross-sectional studies that found HPV16 and HPV6 to be the most prevalent genotypes in MSM populations [13, 17, 22, 23], underscoring the pervasive nature of these infections. Notably, the incidence rates reported in this study were significantly higher in adolescent and young MSM compared with older MSM in China [9, 22, 24], echoing the unique susceptibility of adolescents to HPV infection [25]. This disparity highlights the critical importance of implementing preventative measures, such as HPV vaccination, before sexual debut to effectively reduce the burden of HPV infections in this vulnerable population. In line with these observations, a study conducted in Finland provided compelling evidence supporting the protective efficacy of HPV vaccination in men. Adolescents who received the HPV16/18 vaccine exhibited a profound reduction in the overall HPV DNA prevalence, affirming the vaccine's potential to confer protection against HPV infections [26]. These findings collectively underscore the urgency of prioritizing HPV vaccination strategies tailored for adolescent and young MSM, aiming to curb the high incidence of HPV infections, particularly those involving HR genotypes like HPV16 and HPV6, and ultimately contributing to the prevention of HPV-related diseases in this population.

In our investigation, we noted the persistence of HR HPV genotypes, notably HPV16 and HPV18, which exhibit notably low clearance rates among adolescent and young MSM. This trend mirrors the patterns identified in broader studies, where persistent infection with these oncogenic strains poses a heightened risk for developing HPV-related malignancies over time [27]. According to a previous study, among MSM in China, the persistence rates for HPV16 and HPV18 were notably high, indicating a similar epidemiological concern that aligns with global observations [9]. In contrast, HPV6 and HPV11, associated more with benign conditions like genital warts, were found to clear more rapidly in adolescents [28]. This rapid clearance might underlie the increased incidence of anal warts within this younger demographic, signaling a differential pathogenicity and immune response to various HPV genotypes in adolescent populations [23, 29]. Understanding the distinct clearance patterns of HR versus LR HPV types among adolescent and young MSM underscores the complexity of HPV's epidemiology in this group and highlights the importance of tailored vaccination strategies. Given the nuanced efficacy profile of current HPV vaccines, public health strategies should prioritize vaccine formulations that align with the prevalent HPV genotype distribution and the specific disease burden observed in adolescent and young MSM populations. The insights garnered from our study underscore the imperative for early vaccination interventions to curb the incidence and persistence of HR HPV infections in adolescent and young MSM, thus mitigating the long-term risk of HPV-related diseases. Considering the specific epidemiology of HPV within this group, vaccination strategies should be adaptable and responsive, emphasizing the protection against the most prevalent HPV types to effectively reduce the HPV-related disease burden in this vulnerable population.

Our study identified a significant correlation between the use of online platforms for seeking sexual partners and a heightened incidence of HPV, corroborating findings from earlier studies that have documented the risks associated with digital platforms as venues for initiating sexual encounters [30]. The convenience and anonymity offered by online environments may facilitate engagements in casual sex relationships, often characterized by multiple sexual partnerships and practices that elevate the risk of HPV transmission [31, 32]. Moreover, the advent of geosocial networking applications has further amplified these risks by promoting brief sexual encounters that lack the preventive measures necessary for reducing HPV spread [33]. Casual sex relationships in online settings often involve multiple partners and risky sexual practices, leading to a higher risk of HPV infection [34–36]. Our findings suggest that adolescents with above-average academic performance exhibit lower HPV incidence rates, potentially reflecting the protective effects of higher health literacy and more comprehensive access to healthcare resources [36–38]. This demographic appears more adept at navigating health information and leveraging their knowledge to engage in sexual health practices that minimize their risk of HPV infection. Such behaviors might include the consistent use of condoms, engagement in fewer risky sexual practices, and a more proactive approach to seeking HPV vaccination and screening services. These patterns highlight the critical role of educational attainment in shaping health behaviors and outcomes, suggesting that interventions aimed at improving health literacy could serve as effective tools for reducing HPV transmission among adolescents [39].

The regulatory and technological context of online gay social networks in China differs notably from Western countries. Chinese platforms such as Blued emphasize both synchronous geolocation-based matching and asynchronous messaging, potentially facilitating rapid partner change and casual sexual encounters. In contrast, some Western platforms are more heavily regulated or emphasize privacy features. These contextual differences may influence the frequency and safety profiles of sexual encounters, and our finding of elevated HPV incidence among online sex-seekers may reflect both the individual-level risk behaviors of users and the platform-level features that promote casual partnerships.

Our findings indicated that participants from ethnic backgrounds other than Han Chinese demonstrated a higher rate of HPV clearance. While intriguing, this observation is drawn from a very limited sample—only 2 individuals from non-Han ethnic groups were observed, both of whom cleared their HPV infection. This limitation significantly constrains the generalizability of our results and suggests the need for cautious interpretation. The phenomenon of varying HPV clearance rates across different ethnicities is not well understood and is scarcely documented in the literature, particularly in the context of China's diverse population. Factors that could potentially contribute to differences in HPV clearance rates among ethnic groups include genetic variations, differences in immune response, lifestyle factors, and access to healthcare services, which could influence the likelihood of clearance [40–43]. Given the limited number of participants from non-Han ethnicities in our study, further research involving a larger and more diverse sample is critical to understand the dynamics of HPV clearance across different ethnic groups in China.

The mean age at first anal receptive intercourse (18.7 years) identifies a critical opportunity for early HPV vaccination in Chinese MSM. Current HPV vaccination recommendations in most developed countries suggest vaccination before age 26 years, with greatest benefit if administered before sexual debut. In China, where HPV vaccination is not routinely recommended for MSM, the early onset of sexual debut among young MSM (median age 18 years) indicates that vaccination programs targeting secondary school students (ages 14–18 years) could potentially prevent a substantial proportion of HPV infections in this population. This finding aligns with evidence from other countries demonstrating the cost–effectiveness of prophylactic HPV vaccination in younger MSM cohorts.

Despite these significant contributions, our study has certain limitations that warrant consideration. The relatively long intervals between measurements limited the detection of transient HPV infections, potentially underestimating the true incidence. Additionally, the unforeseen onset of the COVID-19 pandemic introduced significant disruptions to our study's follow-up schedule. The pandemic's impact extended beyond mere logistical challenges, potentially influencing participants’ health-seeking behaviors, sexual practices, and access to healthcare services during this period. These factors collectively might have affected the rates of HPV infection and clearance observed in our study participants during the pandemic. Furthermore, the study relied solely on anal swab testing, potentially missing infections in other anatomical sites. Finally, the lack of updated sociological information during follow-up may have influenced the observed rates of infection and clearance.

The use of the Hybribio HPV detection assay, while validated for genotype-specific detection, has somewhat lower analytical sensitivity than reference methods. This may have led to underestimation of true HPV incidence (missed incident infections at follow-up) and overestimation of clearance (false negatives at follow-up misclassified as clearance). Such bias would generally attenuate associations between risk factors and HPV outcomes, suggesting our risk estimates may represent lower bounds.

Future research should address these limitations by employing more frequent testing, conducting follow-up studies after the pandemic, incorporating testing from additional anatomical sites, and collecting updated sociological data. Additionally, research is needed to explore the underlying mechanisms linking academic performance to HPV outcomes and to evaluate the long-term effectiveness of different HPV vaccination strategies in this population.

## CONCLUSIONS

The high incidence and persistence of HPV infections among adolescent and young MSM in China, especially with HPV16 and HPV6 genotypes, highlight the critical need for focused interventions. It ’s essential to place a premium on HPV-related health education and vaccination outreach within this at-risk group. Active steps, like improving health literacy via educational initiatives and rolling out targeted vaccination campaigns, can substantially reduce the impact of HPV infections and the associated health risks. Adolescent and young MSM could be at the forefront of HPV vaccination efforts to protect their sexual health and overall well-being.

## Supplementary Material

ofag352_Supplementary_Data

## Supplementary Data

Supplementary materials are available at *Open Forum Infectious Diseases* online. Consisting of data provided by the authors to benefit the reader, the posted materials are not copyedited and are the sole responsibility of the authors, so questions or comments should be addressed to the corresponding author.

## Notes

***Acknowledgments.*** The authors thank their partners, including Lingnan (Guangzhou), Pengyou (Foshan), Nanshan Center for Chronic Disease Control (Shenzhen), Chongqing CDC (Chongqing), Tianjin CDC (Tianjin), Shenlan (Tianjin), and Qingai (Qingdao). They thank all participants who made this research possible.

***Ethical approval.*** The UniTest Study was ethically approved by the institutional review boards at Sun Yat-sen University (approval no. SYSU-SPH2018044) and was conducted in accordance with the Declaration of Helsinki. Informed consent was obtained from all participants.

***Data availability.*** The data underlying the findings of this study cannot be made publicly available due to ethical and privacy restrictions, as they contain potentially identifiable information on a sensitive population (adolescent and young MSM) and are subject to the approval of the institutional review board of Sun Yat-sen University (approval no. SYSU-SPH2018044). Deidentified data may be requested from the corresponding author (H. Z.) upon reasonable request and after approval by the ethics committee, provided that the intended use complies with the informed consent obtained from participants. No additional data beyond those presented in this manuscript and its supplementary materials are available.

***Financial support.*** This study was supported by the Natural Science Foundation of China General Program (82574167), Prevention and Control of Emerging and Major Infectious Diseases-National Science and Technology Major Project (2025ZD01905200), National Key Technologies R&D Program of China (2023YFC2306700).
