# Supplementary material for: Incidence, Persistence, and Clearance of Anal Human Papillomavirus Infections Among Adolescent and Young Men Who Have Sex With Men in China: An Observational Cohort Study
Source: Open Forum Infect Dis. 2026 Aug 27;13(8):ofag352. doi: 10.1093/ofid/ofag352 (PMC13521111; doi:10.1093/ofid/ofag352)
Supplement: Supplementary file 1 [file ofag352_supplementary_data.docx]

Supplement Table 1. Demographic characteristics and sexual behaviors of adolescent and young MSM

| Characteristic | Total (n = 426) | Retained in Follow-Up  (n = 200) | Lost to Follow-Up  (n = 226) | *P* |
| --- | --- | --- | --- | --- |
|  | No(%）/Median (IQR) | | |  |
| Age at recruitment (years) |  |  |  |  |
| ≤21 | 225(52.8) | 115(57.5) | 110(48.7) | 0.069 |
| ＞21 | 201(47.2) | 85(42.5) | 116(51.3) |  |
| Ethnicity |  |  |  |  |
| Han Chinese | 408(95.8) | 196(98.0) | 212(93.8) | 0.057 |
| Other | 18(4.2) | 4(2.0) | 14(6.2) |  |
| Study level |  |  |  |  |
| Senior/junior college | 84(19.7) | 35(17.5) | 49(21.7) | 0.337 |
| Bachelor's degree | 342(80.3) | 165(82.5) | 177(78.3) |  |
| Major |  |  |  |  |
| Literature/history | 97(22.8) | 41(20.5) | 56(24.8) | 0.855 |
| Science/technology | 199(46.7) | 96(48.0) | 103(45.6) |  |
| Medicine | 28(6.6) | 13(6.5) | 15(6.6) |  |
| Art | 47(11.0) | 22(11.0) | 25(11.1) |  |
| Kinesiology/other | 55(12.9) | 28(14.0) | 27(11.9) |  |
| Sought sex partner online |  |  |  |  |
| No | 195(45.8) | 95(47.5) | 100(44.2) | 0.410 |
| Yes | 231(54.9) | 105(52.5) | 126(55.8) |  |
| Gay app using |  |  |  |  |
| No | 7(1.6) | 3(1.0) | 4(1.8) | 0.455 |
| Yes | 419(98.4) | 197(98.5) | 222(98.2) |  |
| Recreational drug using sex history |  |  |  |  |
| No | 135(31.7) | 68(34.0) | 67(29.6) | 0.390 |
| Yes | 291(68.3) | 132(66.0) | 159(70.4) |  |
| Drunk sex history |  |  |  |  |
| No | 83(19.5) | 48(24.0) | 35(15.5) | 0.036 |
| Yes | 343(80.5) | 152(76.0) | 191(84.5) |  |
| Ever Heard of HPV |  |  |  |  |
| No | 166(39.0) | 70(35.0) | 96(42.5) | 0.139 |
| Yes | 260(61.0) | 130(65.0) | 130(57.5) |  |
| History of STDs |  |  |  |  |
| Yes | 62(14.6) | 22(11.0) | 40(17.7) | 0.106 |
| No | 288(67.6) | 144(72.0) | 144(63.7) |  |
| Unclear | 76(17.8) | 34(17.0) | 42(18.6) |  |
